# A Technological Review of Wearable Cueing Devices Addressing Freezing of Gait in Parkinson’s Disease

**DOI:** 10.3390/s19061277

**Published:** 2019-03-13

**Authors:** Dean Sweeney, Leo R. Quinlan, Patrick Browne, Margaret Richardson, Pauline Meskell, Gearóid ÓLaighin

**Affiliations:** 1 Electrical & Electronic Engineering, School of Engineering and Informatics, NUI Galway, University Road, H91 TK33 Galway, Ireland; dean.sweeney@nuigalway.ie (D.S.); gearoid.olaighin@nuigalway.ie (G.Ó.); 2 Human Movement Laboratory, CÚRAM Centre for Research in Medical Devices, NUI Galway, University Road, H91 TK33 Galway, Ireland; 3 Physiology, School of Medicine, NUI Galway, University Road, H91 TK33 Galway, Ireland; 4 Neurology Department, University Hospital Galway, H91 YR71 Galway, Ireland; patrick.browne@hse.ie; 5 School of Nursing and Midwifery, NUI Galway, University Road, H91 TK33 Galway, Ireland; 6 School of Medicine, NUI Galway, University Road, H91 TK33 Galway, Ireland; 7 Neurology Department University Hospital Limerick, Dooradoyle, V94 F858 Limerick, Ireland; margaret.richardson@hse.ie; 8 Department of Nursing and Midwifery University of Limerick, Castletroy, V94 T9PX Limerick, Ireland; pauline.meskell@ul.ie

**Keywords:** cueing, Freezing of Gait, FoG, Parkinson’s disease, wearable electronics

## Abstract

Freezing of gait is one of the most debilitating symptoms of Parkinson’s disease and is an important contributor to falls, leading to it being a major cause of hospitalization and nursing home admissions. When the management of freezing episodes cannot be achieved through medication or surgery, non-pharmacological methods such as cueing have received attention in recent years. Novel cueing systems were developed over the last decade and have been evaluated predominantly in laboratory settings. However, to provide benefit to people with Parkinson’s and improve their quality of life, these systems must have the potential to be used at home as a self-administer intervention. This paper aims to provide a technological review of the literature related to wearable cueing systems and it focuses on current auditory, visual and somatosensory cueing systems, which may provide a suitable intervention for use in home-based environments. The paper describes the technical operation and effectiveness of the different cueing systems in overcoming freezing of gait. The “What Works Clearinghouse (WWC)” tool was used to assess the quality of each study described. The paper findings should prove instructive for further researchers looking to enhance the effectiveness of future cueing systems.

## 1. Introduction

Parkinson’s disease (PD) is characterized by four cardinal symptoms (bradykinesia, rigidity tremor and postural instability) occurring due to the degeneration of dopamine-producing neurons [1,2]. One of the most debilitating symptoms of PD is Freezing of Gait (FoG), which is especially present in more advanced stages of the disease [3]. FoG is defined as a “brief episodic absence or marked reduction of forward progression of the feet despite the intention to walk” [4]. FoG is often described by people with Parkinson’s (PwP) as having their feet “glued to the ground” [5]. Three forms of FoG have been identified: a purely akinesia form (no motion of the person’s legs is observed); a ‘‘tremble in place’’ form (inability of the person to step with their legs trembling at a frequency of 2 to 4 Hz) and a ‘‘shuffling’’ form (spontaneous increase in cadence and decrease in step length) [6,7]. Tremble in place and shuffling are the most common forms of FoG, while total akinesia is rare [8]. The presence of FoG is an important contributor to falls in PwP [9,10,11], and is a major cause of hospitalization and nursing home admissions for this group [6,12,13]. This results in a huge personal strain on the PwP, including their carer(s) and substantial economical strain on the health system. Beyond its direct effect on gait, living with FoG has additional PD associated non-motor complications such as anxiety, social isolation and depression and dramatically affects the person’s quality of life (QoL) [14,15,16,17,18,19].

The pathophysiology of FoG is complex and unclear, and despite limited understanding of the disrupted physiological processes associated with FoG, a number of treatment options are currently available. However, clinical consensus on a cohesive treatment protocol for FoG does not exist [20,21]. FoG can also be categorized based on its responsiveness to dopaminergic medication (Table 1) [22]. Although no large-scale studies on the prevalence of dopaminergic-responsive FoG were reported, an observational multi-center study by Amboni et al. described 61.6% of 325 PwP with FoG that only occurred in the off-medication state [23]. The primary treatment option for dopaminergic-responsive FoG is to increase dopaminergic medication [3]. However, for some PwP, increasing the dose of dopaminergic medication is often complicated by dose-limiting side-effects, such as dopaminergic-induced dyskinesia [24]. Under these circumstances, surgical procedures such as pump-delivered therapies and deep brain stimulation (DBS) of the subthalamic nucleus (STN) may become an effective treatment option [21].

Dopaminergic-induced FoG is uncommon and in the case of Amboni et al. only 1.8% of the 325 PwP had FoG that only occurred in the on-medication state [23]. The primary treatment option for dopaminergic-induced FoG is to reduce dopaminergic medication [3]. However, the reduction of dopaminergic medication is impossible for some PwP, due to unacceptable worsening of the cardinal symptoms of PD. Under these circumstances, DBS of the STN may again become an effective treatment option [21]. However, this treatment option does not directly improve dopaminergic-induced FOG, but only alleviates the problem indirectly by treating the cardinal symptoms of PD, enabling a reduction in the dopaminergic medication. No large-scale studies on the prevalence of dopaminergic-resistant FoG were reported, however, Amboni et al. described 36.6% of 325 PwP had FoG in the on-medication and off-medication states [23]. Treatment options for dopaminergic-resistant FoG are primarily in the research setting and are not currently implemented in clinical practice. Limited evidence suggests that Levodopa-carbidopa intestinal gel therapy [25]; transcranial direct current stimulation (tDCS) [26]; and DBS of the pedunculopontine nucleus (PPN) [27] may become possible treatment options, however, further research is required if they are to be clinically accepted.

The above-mentioned treatment options for FoG have some limitations: (1) changing dopaminergic medication to manage dopaminergic-responsive or dopaminergic-induced FoG can result in dose-limiting side-effects or worsening of the cardinal symptoms. (2) Surgical options are invasive and costly ($35,000 to $50,000 for DBS (National Parkinson’s Foundation) Current research-based treatments for dopaminergic-resistant FoG are lacking clinical evidence. Consequently, there is a need to provide new and improved treatment options for FoG. One such treatment option is the adoption of cueing techniques.

Cueing is a well-established technique that has been shown to improve gait in PwP including increasing walking speed, step length, cadence (total number of steps taken per minute) and reducing the number of FoG episodes [2,28].

Cueing can be defined as using external stimuli which provides temporal (related to time) or spatial (related to space) information to facilitate movement (gait) initiation and continuation [29].

The effect of cueing modalities on the specific subtype of FoG (i.e., dopaminergic-responsive, dopaminergic-induced or dopaminergic-resistant FoG) is poorly reported in literature. This may be due to the fact that distinguishing the different FoG subtypes requires a comprehensive motor assessment in at least three medication states [30]. It is more common for the literature to report the effect of cueing modalities on FoG that occurs while the PwP is in either; (1) the on-medication state (referred to as On-FoG); (2) the off-medication state (referred to as Off-FoG) or (3) the end-of-dose-medication state (referred to as EoD-FoG).

Three cueing modalities are extensively reported in literature: visual cueing, auditory cueing and somatosensory cueing. These modalities reflect the specific external stimuli that the cueing technique utilizes (i.e., visual cueing provides visual stimuli). A further modality may be considered when combining two types of cueing modalities (i.e., visual-auditory cueing simultaneously provides both visual and auditory stimuli).

The precise mechanism(s) underlying the effectiveness of cueing to ameliorate FoG is unclear, however, previous studies have suggested:

1. Cueing may compensate for the defective internal rhythm generator of the basal ganglia, consequently affecting the coordination and execution of movement [31,32]. In this way, the PwP may use auditory, visual or somatosensory cueing to provide temporal information (external rhythm) to which movement can be coupled [33].
2. Another theory is that the PwP may use visual cueing to provide spatial information to scale and guide movements, which may allow the PwP to bypass their defective basal ganglia during gait [34].
3. Previous studies also have suggested that cognitive/attentional mechanisms might explain the positive effects of cueing on FoG. Namely, auditory, visual or somatosensory cueing may shift the PwP’s attention to the task of walking, helping them to consciously think of what to do next [35].
4. Studies indicate that enhanced proprioceptive information processing could be the mechanism underlying the positive effects of cueing on FoG. In this way, the PwP may use visual or somatosensory cueing as an artificial means to stimulate the proprioceptive inputs, providing enhanced information on limb position and movement during gait [36].

**Figure 1:**
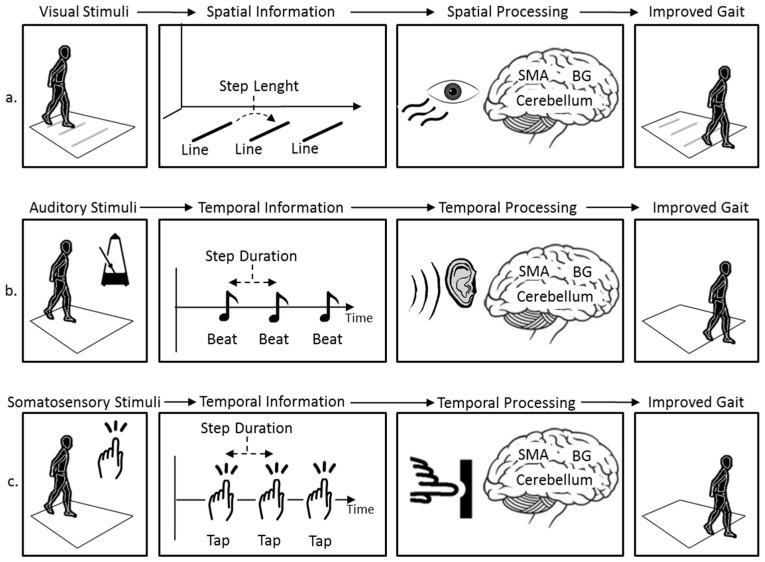
Three cueing modalities to ameliorate FoG. (**a**) Visual cueing: parallel lines marked on the ground at a fixed distance apart, conveys spatial information, such as step length; (**b**) Auditory cueing: a metronome producing auditory stimuli at a set beat, conveys temporal information, such as step duration; (**c**) Somatosensory cueing: Tapping of a people with Parkinson’s (PwP) shoulder at a set rhythm, also conveys temporal information, such as step duration. Both spatial and temporal information may be perceived by PwP and processed through multiple brain regions (BG—basal ganglia, SMA—supplementary motor area and cerebellum) to ameliorate FoG and improve gait.

The use of visual cueing to ameliorate FoG was reported as early as 1990 [37]. Dietz et al. demonstrated that walking over parallel lines marked on a floor (visual stimuli) could reduce the occurrence of FoG. Parallel lines can provide a simple method of conveying spatial information, such as step length, through the spacing of each line (Figure 1a). For instance, Dietz et al. spaced parallel lines every 305 mm and instructed the PwP to step over each line as they walked across the floor, thus regulating their step length. It is unknown if the action of regulating the PwP’s step length directly results in a reduction in the number/duration of FoG episodes or if the simple method of drawing attention to the stepping process (i.e., thinking about stepping over the lines) was the contributing factor. Or indeed, if visually focusing on the lower limbs during the stepping process (providing enhanced proprioceptive information) was a contributing factor.

Auditory cueing was reported to ameliorate FoG in a study by Enzensberger and colleagues in 1997 [38]. In this paper, Enzensberger proposed the use of a metronome to investigate its effect on FoG. Metronomes are devices that provide a regular, repeated audible sound (i.e., tick, beat and click) at an interval that can be adjusted, typically in beats per minute (bpm). They provide a simple method of conveying temporal information, such as step duration, through the rhythmical timing of each beat (Figure 1b). For instance, Enzensberger et al. set the rhythm of the metronome to 95 beats/minute and instructed the PwP to step in time to the beat of the metronome, therefore modifying and regulating the PwP’s step duration to 0.63 s. In addition, Enzensberger et al. also proposed the use of shoulder tapping (somatosensory stimuli) to investigate its effect on FoG [38]. Shoulder tapping can convey temporal information, such as step timing, through rhythmically tapping on the PwP’s shoulder (Figure 1c).

Initial reviews by Lim et al. in 2005 and Nieuwboer et al. in 2008 on cueing, suggested insufficient evidence for the effectiveness of cueing to ameliorate FoG [2,35]. However, only six studies in total were included in the reviews [29,37,38,39,40,41], possibly reflecting the limited number of published studies investigating cueing as a method to ameliorate FoG prior to 2008.

Over the last decade, a significant number of studies, investigating the effect of cueing on FoG, have been published. These studies can be grouped into two categories.

- Immediate cueing: the majority of studies have investigated the immediate effect of cueing on FoG [36,42,43,44,45,46,47,48,49,50,51,52,53,54,55,56,57,58,59]. These studies predominantly evaluated the effect of cueing over a single session of use, with the objective of the cueing being to provide an immediate benefit in terms a reduction in the number/duration of FoG episodes.
- Therapeutic cueing: a number of studies have investigated the therapeutic effect of cueing [60,61,62,63,64,65,66,67,68,69,70]. During these studies, the PwP engaged in a number of therapy sessions per week (treatment period). The cueing in this case was only delivered during the therapy sessions. These therapy sessions aimed to provide a therapeutic benefit, which ameliorated FoG when the person was walking at home or in the community without any cueing being provided.

These different approaches to ameliorate FoG have provided various technical challenges that led to the development of novel cueing systems. Examples of these cueing systems are custom-built metronomes [44], laser light canes [42], optical head-mounted displays [56], custom-built vibratory units [36] and electrical stimulation systems [54]. The effectiveness of these systems to ameliorate FoG has been suggested to be strongly dependent on the cueing modality adopted and the specific information that the cue provides [35]. Additionally, a key challenge identified by Lim et al. [2] is whether positive laboratory-based results translate into similar performance in daily living environments. Therefore, to enhance our understanding of the technical characteristics of current cueing systems and the effectiveness of these systems, this paper aims to present a chronological review of the literature relating to the development and performance of cueing systems. This technological literature review may prove instructive for future researchers looking to enhance the effectiveness of this modality and may serve to further inform the development of cueing systems for take-home applications.

## 2. Methods

### 2.1. Review Questions

A technological review of the literature on cueing devices addressing FoG in PD was carried out in an attempt to address the following research question: what is the current state of the art in cueing systems that have the potential to be used at home as a self-administer intervention for FoG and how effective are they?

### 2.2. Article Search Strategy

Article selection was based on a search for publications following the PRISMA guidelines [71] of the following scientific databases: IEEE Xplore, Embase, PubMed, Science Direct, Scopus, and Web of Science. All publications from January 2009 to December 2018 were included in the search. The electronic databases were searched by the first author (D.S.) for relevant articles using the following search terms mentioned in the title or abstract or keywords: “Parkinson” AND (“cue” OR “cues” OR “cueing” OR “freezing”).

In addition to the search terms, the following search filters were applied: (i) full journal article; (ii) written in English; (iii) appeared in a peer-reviewed academic source. To identify additional articles, a snowball technique was used through additional hand-searching of references and citation tracking from relevant articles.

### 2.3. Article Screening

Articles were initially screened by the first author (D.S.) based on title and abstract. Articles were excluded from the systematic review if they fulfilled any of the following criteria: (i) did not present a wearable cueing system to address FoG in PD; (ii) systems could not be used for take-home applications (ii) present insufficient technical detail on the cueing system (iii) not full journal article or reported on previous published work and (iv) written in any language other than English. Where the title or abstract did not provide sufficient detail to determine exclusion, full-text articles were reviewed. Based on the above criteria, full-text revisions were performed by the first author (D.S.) and then assessed independently for eligibility by the second authors (G.OL. and L.R.Q.).

### 2.4. Quality Assessment

The studies included in this review primarily used single-case design approaches. These types of studies involve within-subject repeated measures and can provide a viable alternative to large group studies. Quality assessment tools for single-case studies are relatively new, with no currently accepted “gold standard” tool [72]. However, a well-established tool that has been recommended for use in systematic reviews of single-case studies, is the “What Works Clearinghouse (WWC)” tool [73]. This tool assesses the validity of single-case studies, classifying them as either “Meeting Standards,” “Meeting Standards with Reservations,” or “Not Meeting Standards” [74]. As described in the WWC guidelines, studies were required to describe the systematic manipulation of the independent variable with the researcher actively determining when and how experimental conditions were changed. Upon successful demonstration of control of the independent variable by the researcher, studies were then further assessed as follows:

- Each outcome variable was systematically measured and an inter-observer agreement (IOA) was reported for a minimum of 20% of data for each experimental condition. The IOA score must meet or exceeded 80%.
- The study included at least three replication attempts of intervention effects at different points in time or in three different phase repetitions.
- An intervention phase met the minimal data point threshold for the design type as specified by WWC guidelines [74].

## 3. Results

**Figure 2:**
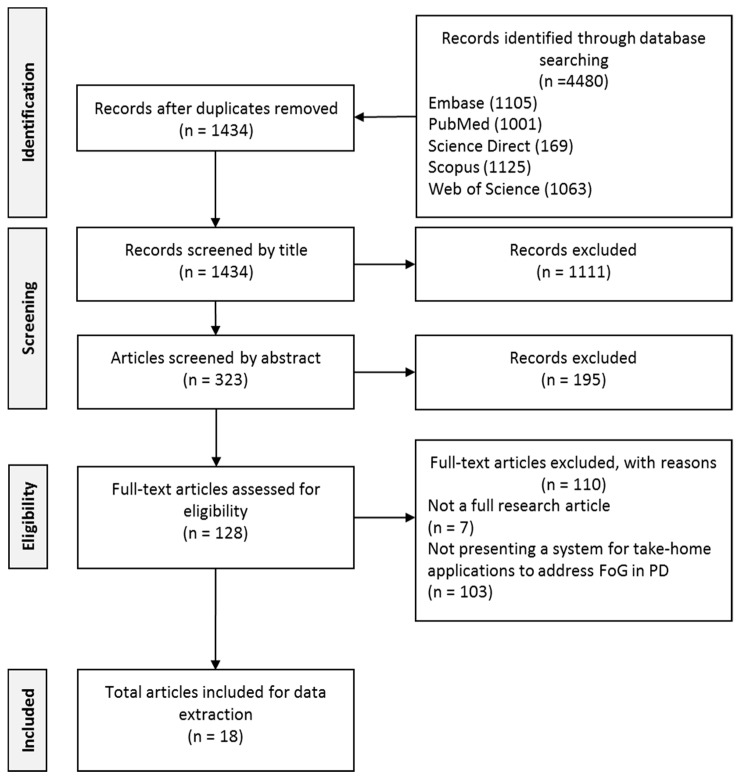
PRISMA flow diagram of study selection.

The outcome of the systematic search is summarized in Figure 2. The initial search identified 4480 records. Reference manager software EndNote X5 (Thomson Reuters, Philadelphia, PA, USA) was used to collate results. Duplicates were removed and a screening process of both the title and abstract of the remaining records was subsequently conducted. The full-text of the remaining records were then assessed for relevance to the review. Following this procedure, 18 articles remained for the systematic review.

### 3.1. Auditory Cueing Devices

**Figure 3:**
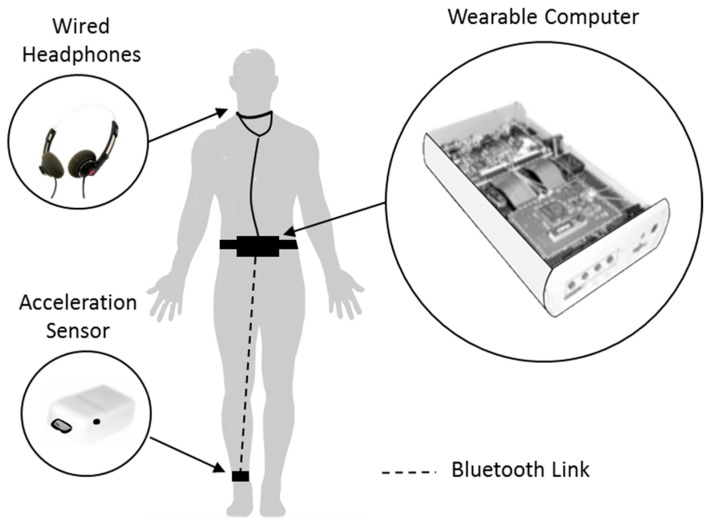
Illustration of user wearing the On-demand auditory cueing system, presented by Bachlin et al. A wired headphone was worn around the neck of the user and connected to a wearable computer that was attached to the lower back. An acceleration sensor was attached to the shin and connected to the wearable computer over a Bluetooth classic communication link.

In 2010, Bachlin and colleagues identified limitations with metronome cueing devices, such as those previously reported by Enzensberger et al. and Cubo et al. [38,39]. When activated, these devices continually delivered auditory cueing regardless of whether FoG was present or not. This design limitation was identified by Cubo et al. who commented on the possible problem of continuous auditory cueing, reporting that PwP may become habituated to the auditory stimuli, thus reducing the effect of cueing [39]. To address this limitation, Bachlin and colleagues presented an auditory cueing system that provided auditory cueing only during an actual FoG episode [75,76]. In comparison to a continuous cueing system, which aims to prevent FoG from occurring through continues cueing, Bachlin’s system provided On-demand cueing which proposed to relieve FoG once it happened. The system was implemented using a wearable research platform designed for rapid prototyping (Figure 3). The platform provided a custom wearable computer that interfaced to sensors and the user. On a 3.3 Ah battery, the wearable computer ran for more than 6 h. In its then configuration, wired headphones were placed around the user’s neck and connected to a small wearable computing (132 × 82 × 30 mm^3^ and 231 g) worn on the waist. An acceleration sensor (22 × 41 × 12 mm^3^ and >22 g) was attached to the shank (just above the ankle) of the user and transmitted data to the small wearable computer over a Bluetooth classic communication link. Seminal work carried out by Hausdorff et al. [7]; and Moore et al. [77]; into the principles of FoG detection algorithms enabled Bachlin and colleagues to develop a FoG detection algorithm for real-time operation. The algorithm was implemented with a lag time (time between the occurrence of a FoG episode and its detection) of less than 2 s. The main computational part of the algorithm used an fast Fourier transform (FFT) calculation to determine the power content of the acceleration sensor data between 0.5 and 3 Hz (walking band) and 3 and 8 Hz (freeze band), as suggested by Moore et al. [77]. A method of thresholding was used to determine if the user was in a standing, walking or FoG state. By implementing their algorithm within the Linux operating system of the small wearable computer, the system would produce a rhythmic ticking sound at a tempo of 60 beats/minute whenever a FoG episode was identified, and this lasted until the user resumed walking. For all users, this provided a cue every one second, during the period the user was in FoG. Bachlin et al. did not explain why they choose to use auditory cueing with a fixed tempo of 60 beats/minute. Interestingly, the normal cadence for men and women aged 65–80 years was reported as, 81–25 steps/minute and 96–136 steps/minute respectively [78]. This was significantly higher than the 60 beats/minute produced by the auditory cueing system.

In a repeated measures proof of concept study, Bachlin and colleagues carried out measurements on 10 participants (eight in the On-medicated state and two in the Off-medicated state) to understand the technical effectiveness of their system and to collect feedback about the system. During laboratory-based waking tasks both the accuracy of the online FoG detection algorithm and the effectiveness of the system at reducing the frequency and duration of FoG episodes was evaluated. Using a subjective questionnaire, Bachlin et al. presented data on the experience of eight participants (two of the 10 participants did not experience FoG during walking tasks). Three participants expressed no change in frequency of FoG episodes and five expressed a lower frequency of FoG episodes with the cueing system compared to without the system. In addition, two participants expressed no change in the duration of FoG episodes; five expressed shorter FoG episodes and one expressed longer FoG episodes with the system compared to without the system. However, no statistical data was presented on the frequency or duration of FoG episodes during the walking tasks. Therefore, no statistical conclusion could be made on the systems effectiveness in ameliorating FoG. Furthermore, Bachlin and colleagues acknowledged the short time available for the participants to test the system (<1 h) and were only in a position to speculate on what might be the benefit of the system over a longer duration. A drawback of the system was the varied level of sensitivity and specificity of the online FoG detection algorithm among participants, with average values of 73.1% and 81.6%, respectively. For some participants, the low specificity value resulted in the participants requesting to hear the auditory cueing less and for some participants, the low sensitivity value resulted in the participants requesting to hear the auditory cueing more often.

This was reported by Bachlin et al. to affect the overall acceptance of the system. Although Bachlin et al. showed that a user-specific optimization of the FoG detection algorithm could improve sensitivity and specificity to 88.6% and 92.4%, this was demonstrated during offline post-processing.

It is worth noting that, although the system was bulky, only one participant commented that it should be miniaturized. Participants also commented negatively on the use of the headphones around the neck, reporting “that people in the surroundings should not notice they are wearing such a system, either by seeing it or by hearing the ticking sound”. One participant suggested introducing variations in the audio tone and rhythm to avoid becoming used to the system. Bachlin et al. also presented data on the feedback of four physiotherapists on the system. Two physiotherapists saw potential in the system to support PD participants in their everyday life, although, two thought it less suitable, especially due to the size and the attachment method to the belt. However, the physiotherapist did not see any indication that the size of the system disrupted the participant’s normal gait. One physiotherapist suggested adjusting the tempo of the auditory cueing according to the cadence of the participant. This was an important comment, as previous work had identified that when different tempi were utilized, auditory cueing changed some of the altered walking parameters (stride length, cadence and gait velocity) in PwP [79].

Interestingly, Bachlin et al. also investigated multiple on-body locations for the optimal location of the acceleration sensor during the trial; shank (just above the ankle), and thigh (just above the knee of the same leg) and the trunk of the participant. Offline comparative analysis of these on-body sensor locations showed little difference in the accuracy of the FoG detection algorithm, indicating that an acceleration sensor may be placed at multiple locations without losing significant accuracy. However, Bachlin et al. reported that the best result was achieved when using the vertical axis of the acceleration sensor located at the knee and the worst was achieved when using the vertical axis of the acceleration sensor located at the hip. Bachlin et al. commented that performance may be further enhanced by using multiple sensors, especially for users that do not exhibit trembling of both legs during FoG. In conclusion, Bachlin et al. acknowledged there were further possibilities for technical improvements. These improvements included; (1) the integration and miniaturization of the system into a single sensor node and (2) adjustment of the auditory cueing tempo using sensor recordings of the user’s natural cadence.

In the same year, Arias and Cudeiro presented a custom-built portable device that provided auditory cueing via a set of headphones [44]. In this paper, Arias hypothesized that auditory cueing delivered at a tempo scaled to the user’s cadence (10% above the normal cadence of the user), would modify their walking pattern and help to alleviate EoD-FoG. To investigate the hypothesis Arias et al. developed an auditory cueing device that provided a rhythmic 4625 Hz auditory tone, delivered in bursts of 50 ms duration with an adjustable tempo. The functionality of the system lacked the sophistication of Bachlin’s system, by comparison, did not provide auditory cueing when required but instead provided continuous auditory cueing at a tempo scaled to the user’s cadence. Implementing an adjustable tempo was an important feature of Arias and Cudeiro device, as the ideal cueing tempo to improve gait in PD is now receiving widespread attention [50,80]. Within the paper, the authors did not explain the choice of a 4625 Hz tone, delivered in pulses of 50 ms, but it may reflect that humans are most sensitive to low-intensity sounds at frequencies between 2000 and 5000 Hz.

To investigate the effect of their system on EoD-FoG, Arias and Cudeiro carried out measurements on 10 participants as they performed a ~16 m walking task with and without auditory cueing. Video footage analysis from each waking task was performed by a specialist, who identified the frequency and duration of FoG episodes. Arias and Cudeiro observed a significate immediate positive effect of auditory cueing on the mean frequency of EoD-FoG episodes (without cueing: 5.9 ± 6.707; with cueing: 1.4 ± 1.265, *p* = 0.014) and mean duration of EoD-FoG episodes (without cueing: 3.119 ± 4.93 s; with cueing: 1.02 ± 1.699 s, *p* = 0.017). However, the study failed to meet WWC single-case design standards due to a lack of dual assessor’s analyses of outcome measures. Furthermore, Arias and Cudeiro acknowledged the short time provided to the participants to test the device, and therefore its efficacy during repeated, daily use is not reported.

**Figure 4:**
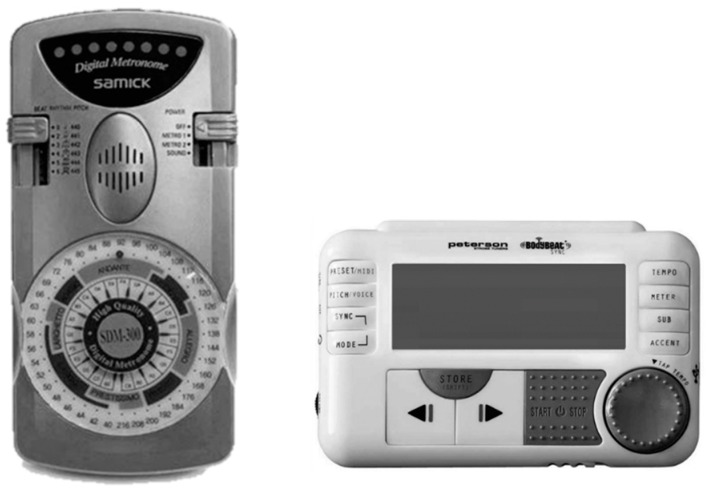
Both the SDM300 SAMICK and Peterson BodyBeat Pulsing Metronome provide features such as tempo, tone and volume adjustments, a built-in speaker and a headphone jack.

Following Arias and Cudeiro’s work, research attention shifted from the development of custom-built wearable auditory cueing devices to the utilization of technologies available in the market-place. In 2012 and 2016, Lee et al. and McCandless et al. respectively investigated the effect of rhythmic auditory cueing on FoG using commercially available electronic metronomes [45,50]. The SDM300 SAMICK Metronome (SAMICK Musical Instruments Co., Ltd., Enmseong County, South Korea), featured two large tempo and tone control knobs (40–216 beats/minute and 45–440 Hz tone), a built-in speaker and a headphone jack. The device was comparable in size to Bachlin’s research platform, but it did not feature a means to attach it to the user (Figure 4).

While investigating the immediate effect of auditory cueing on Off-FoG, Lee et al. carried out measurements on 15 participants with FoG as they performed a ~14 m walking task without auditory cueing, with auditory cueing and with visual floor markers. Based on previous results (which investigated the effect of tempo on stride length and walking speed of PwP in the On-medicated state) [81], and unlike Arias and Cudeiro, Lee et al. choose to set the tempo of their device from 10% to 20% below the normal cadence of the participant. Lee et al. reported a significant immediate negative effect of auditory cueing on the mean frequency of Off-FoG episodes (with cueing: worsened by 56.7 ± 8.2%, *p* = 0.024). Although, video footage was analyzed for outcome measures by two assessors, inter-assessor agreement was not documented using a statistical measure, therefore, this study failed to meet WWC single-case design standards. It was unclear if headphones or the speaker was used during the study as Lee et al. did not provide information on the setup of the metronome. Again, user experience results were not reported, which could have provided insight into the effectiveness of commercial metronomes, during everyday use, to manage FoG.

The BodyBeat Pulsing Metronome (Peterson Electro-Musical Products, Inc., Alsip, IL, USA), featured a digital LCD, a keypad control, a built-in speaker and a headphone jack (Figure 4) [45]. The device is smaller in size to the SDM300 metronome and provided a clip for attachment to the user. The auditory cueing tempo could be adjusted between 10 and 280 beats/minute, and the device could produce four different metronome rhythmic sounds (Rimshot, Clave, Wood Block and Beep).

To investigate the immediate effect of auditory cueing on Off-FoG, McCandless et al. carried out measurements on 20 participants as they performed a >3 m walking task with and without auditory cueing. McCandless et al. set the tempo of their device to either 70, 60 or 50 beats/minute, based on participant’s preferences.

McCandless et al. reported an immediate positive effect of auditory cueing on the mean percentage of Off-FoG episodes during the walking task (without cueing: 81.58 ± 7.53%; with cueing: 44.44 ± 7.74%). Inter-assessor agreement was not documented, therefore, this study failed to meet WWC single-case design standards. As in Lee’s case [50], McCandless did not provide information on the setup of metronome or user experience results.

**Figure 5:**
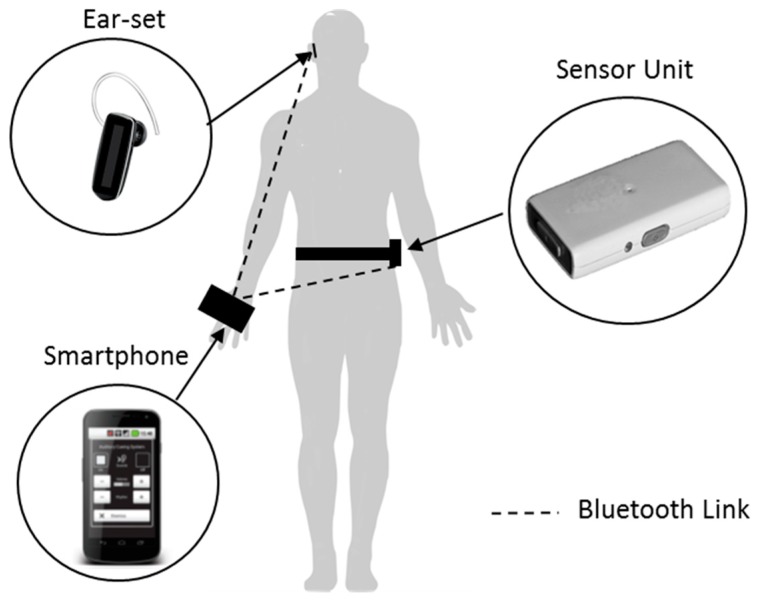
Illustration of user wearing the auditory cueing system, developed by Sama et al. Wireless Samsung (Samsung Group. Seocho District, Seoul, South Korea) HM3500 ear-set, a sensor unit around the user’s waist and a smartphone (Samsung Galaxy Nexus) with auditory cueing application was worn on-body. The smartphone connected to the ear-set and sensor unit over a Bluetooth classic communication link.

In 2014, research attention shifted from laboratory-based evaluations to home-based evaluations, and with it, the development of auditory cueing devices designed for PwP and take-home applications. In this year, Samá et al. presented the REMPARK system [82]. The system was developed as a personal health device for the remote and autonomous management of PD and included an auditory cueing system developed to improve gait and relief FoG. Although Bachlin et al. previously proposed that their auditory cueing system could be miniaturized into a single sensor unit that could detect FoG and transmit a trigger signal to a feedback device [75], Samá et al. were the first to present such a system (Figure 5). Samá et al. presented a custom-built single sensor unit (77 × 37 × 21 mm^3^ and 78 g) that attached to the trunk of a user. The sensor unit implemented frequency analysis and machine learning approaches to determine the presence of PD symptoms such as bradykinesia, dyskinesia, and FoG. The main reference for the algorithmic approach is described by Ahlrichs et al. [83]. Upon detection of a symptom(s), the sensor unit would transmit a trigger signal to the feedback device (smartphone), which streamed rhythmic auditory cueing to an ear-set until the user resumed walking without FoG or bradykinesia.

To increase acceptability and reduce possible stigmatization, Samá et al. adopted a smartphone as the feedback device. This refinement in the design of auditory cueing systems enabled the development of smartphone application interfaces that were specifically tailored to the needs and abilities of PwP. In a further follow-on publication, Nunes et al. presented detailed information on the development of the auditory cueing smartphone application [84]. In summary, the smartphone application allowed the user to select their preferred auditory cueing settings: turn on/off auditory cueing, change volume, change the type of auditory stimuli (metronome sounds, musical beats, clapping and verbal cueing) and adjust the tempo (beats/minute). Another refinement in the design of auditory cueing systems was the use of a single wireless ear-set. This refinement enabled the users not only to hear the auditory cueing but also to perceive other auditory stimuli from the surrounding environment.

Unfortunately, Samá et al. did not perform a clinical study to evaluate the effectiveness of the system to ameliorate FoG. However, Samá et al. reported that the FoG detection algorithm could be employed with a lag time of roughly 3.2 s (time between the appearance of a FoG episode and its detection) with a sensitivity of 82.2% and specificity of 92.8%. The long lag time may prove to be a drawback of the system as it reduced the applicability of the system to provide auditory cueing for FoG that persists for less than 3.2 s.

**Figure 6:**
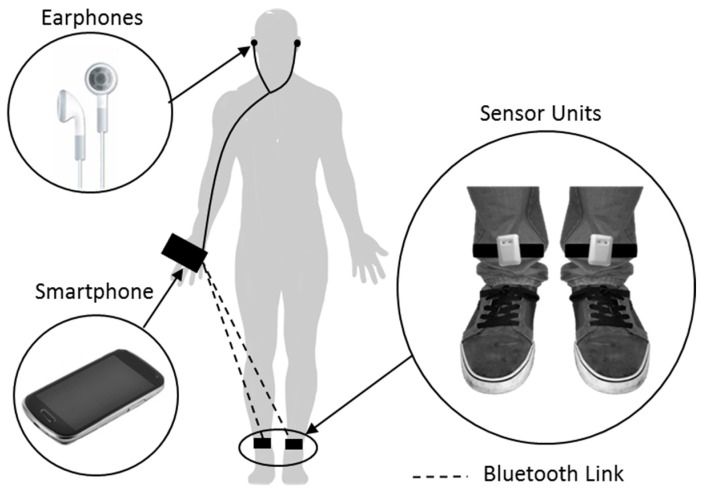
Illustration of a user wearing the GaitAssit system. Wired earphones were placed in the user’s ear, two sensor unit are attached to each lower leg and a smartphone (Samsung S3 mini) with auditory cueing application was worn on-body. The smartphone connected to each sensor unit over a Bluetooth classic communication link.

In 2015, Mazilu et al. presented the GaitAssist system, an auditory cueing system developed to provide motor training and gait assistance at home [85]. The GaitAssist system was similar to the Samá et al. auditory cueing system. However, Mazilu and colleagues presented further refinements to the design of an auditory cueing system. In comparison to the Samá et al. auditory cueing system, Mazilu et al. adopted a smartphone as the feedback device and also utilized the computing capabilities of a smartphone to perform FoG detection. Mazilu et al. developed a smartphone application that implemented frequency analysis and machine learning techniques to detect FoG and produce a rhythmic ticking sound when FoG was identified or anticipated. Based on prior works investigating the optimal sensor location for the detection of FoG [75,86], Mazilu et al. presented two custom-built sensor units that attached to each ankle of a user (Figure 6). By streaming movement data from these sensor units in real-time to the smartphone, Mazilu et al. demonstrated that their FoG detection algorithm could be employed with a lag time of approximately 0.5 s at a sensitivity of 97.1% and a false negative rate of 26.5%. The reduced lag time was in part due to employing a processing data window of 2 s, while Bachlin et al. and Sama et al. used a window of 4 and 3.2 s, respectively.

In addition, the smartphone application could be used to automatically customize the auditory cueing tempo to the user’s cadence through real-time analyses of gait data. This method was an innovative step that enabled a fast, robust and straightforward setup of the auditory cueing tempo. The system performed automatic customization of the auditory cueing tempo by requesting the user to walk naturally in a straight line, for 30 s, between a “Start” and “End” signal given by the smartphone. Following the request, the system would analyze the movement data from the two sensor units and automatically count the number of steps and compute the cadence of the user.

In a proof of concept study, Mazilu et al. carried out a three day study on five participants which assessed the immediate effect of their system on FoG during natural daily life activities. Results showed a reduction in the tread of FoG frequency and durations for four out of five participants over the course of the three days. However, the analysis was made on sensing data from five participants, and therefore no statistical conclusion could be made on the system’s effectiveness in ameliorating FoG. Interestingly, Mazilu et al. evaluated the performance of the system using the FoG detection capabilities of the system. The system stored the frequency and duration of FoG episodes detected during the three days of study. Moreover, as highlighted by Moore et al., wearable sensing data is considered a valid objective method of assessing FoG [77]. This method of evaluation reduces the complexity of performing home evaluations, which would require video recordings (for gold-standard measurements) and provoke privacy issues.

Using a subjective questionnaire, Mazilu et al. also presented data on the experience of nine participants who used the system for approximately three hours in total over a period of one week. Mazilu et al. observed one common wearability drawback of the system; participants reported difficulties in attaching the sensor units, requiring help from another person. Participants also commented on the use of the earphones, and although, there were no reported issues with plugging the earphones into the smartphone’s socket, participants preferred not to use them because they could also hear the auditory stimuli through the mobile phone’s speaker. Overall, participants presented a positive opinion toward using the system in daily life.

**Figure 7:**
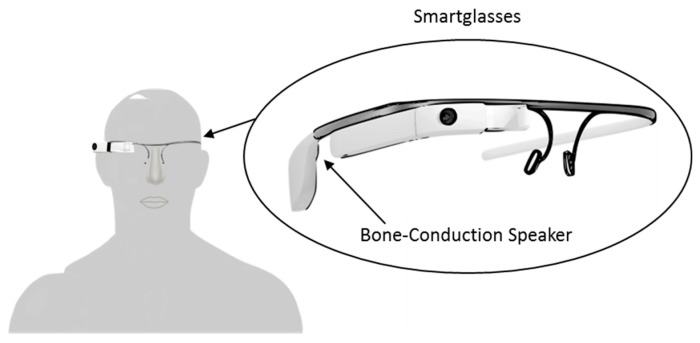
Illustration of the continuous auditory cueing system, used by Zhao et al. The Google ‘Glass’ system provided features such as monocular display and bone-conduction sound. During auditory cueing the monocular display is clear and the bone-conduction speaker delivers a burst of audio cueing at a set tempo.

In 2016, following the introduction of smartglasses by Google (Google LLC. Mountain View, California, USA) and Microsoft (Microsoft Corporation. Redmond, Washington, USA), Zhao et al. suggested that this new technology may make cueing more accessible to PwP [48]. Smartglasses share similar features with smartphones (e.g., GPS, WiFi, accelerometers, and audio/visual output), however, unlike smartphones their displays can be conveniently worn like conventional glasses. To investigate the feasibility of smartglasses as cueing devices, Zhao et al. proposed to use Google Glass as a platform to evaluate the effect of continuous auditory cueing on FoG. Zhao and colleagues developed an Android app for Google Glass that utilized the bone-conduction and voice/gesture control capabilities of Google Glass (Figure 7). Bone-conduction allowed users to hear the rhythmic auditory sound through vibrations of Google Glass.

Users interfaced with the app through voice/gesture control which allowed the user to adjust the auditory cueing tempo between 50 and 150 beats/minute.

To investigate the immediate effect of auditory cueing on EoD-FoG, Zhao et al. carried out measurements on 12 participants. During laboratory-based, 20 and 8 m walking tasks, the frequency and duration of EoD-FoG episodes were evaluated with and without auditory cueing. In line with previous research [46], Zhao et al. chose to set the tempo of their device to the normal cadence of the participants. Zhao et al. reported no significant changes in the frequency and duration of EoD-FOG episodes when all walking tasks were considered together. However, Zhao et al. observed a significant immediate effect of auditory cueing on the mean frequency of EoD-FOG episodes (*p* < 0.05) during walking tasks with 360° turning. This study meets WWC single-case design standards, however due to the small sample size of FoG episodes (six participants experienced EoD-FoG more than once, four exhibited no EoD-FoG, and two had a single EoD-FoG episode), the effect of auditory cueing on EoD-FoG was reported to be inconclusive.

Using a subjective questionnaire, Zhao and colleagues presented data on the experience of participants who were tested with the device. Nine participants were willing to use Google Glass at home to address FoG. Seven participants reported they found Google Glass easy or very easy to use and nine participants reported the instructions on the screen clear or very clear to read. One participant particularly liked the bone-conducting headphone because the metronome was less audible to others around them. Zhao et al. acknowledged there were further possibilities for technical improvements before Google Glass can be adopted for daily use, which included the ability to detection FoG.

### 3.2. Visual Cueing Devices

**Figure 8:**
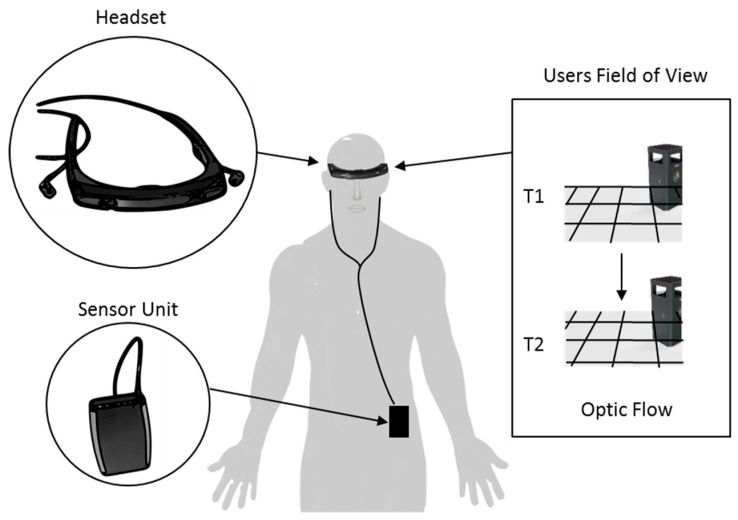
Illustration of the Visual-auditory walker, developed by Espay et al. The system was composed of a sensor unit and a head-mounted micro-display with earphones. The sensor unit was attached to the user’s clothing and the headset worn around the eyes. The system provided continuous optical flow visual cueing. During cueing, a virtual checkerboard-tiled floor is overlayed on top of the user’s field of view (T1) and continually moves forward on the screen at a rate equal to the user’s real-time gait speed (T2).

In 2010, Espay et al. highlighted that cueing systems predominantly provided cueing at a set tempo that was scaled to the user normal cadence [61]. However, preliminary clinical studies indicated that when the tempo of cueing was not externally set, but, was an outcome of the user’s current motion (i.e., dynamic), PD gait could be improved [79,87]. To investigate this hypothesis, Espay et al. developed the Visual-auditory walker, a continuous visual-auditory cueing system for home-based use by PwP [61]. The system was composed of a sensor unit and a head-mounted micro-display with earphones (Figure 8). The system simulated optic flow (pattern of apparent motion in a visual field of view) by displaying a virtual checkerboard-tiled floor superimposed on the real world. The virtual checkerboard-tiled floor responded dynamically to the user’s motion and moved toward them at a speed set equal to their gait speed, as measured by the sensor unit. The checkerboard-tiles provided targets for the user to step on with long strides as they walk. In addition to the visual display, the system also offered auditory feedback from the user’s steps, through earphones.

In a feasibility study, Espay et al. carried out measurements on 15 participants to assess the effect of optical flow visual cueing on FoG over a two weeks treatment period. During the reported study, participants were instructed to use the visual cueing system while walking for no less than one hour a day. Before and after the treatment period, a FoG questionnaire (FOGQ) [88] was used to evaluate the effect of visual cueing on FoG. The FOGQ is a validated clinical tool that uses a six-item questionnaire to assess the severity of FoG in everyday life subjectively. The questionnaire scores the severity of FoG from 0 to 24, with the higher scores corresponding to more severe FoG. 

Over the two weeks treatment period, Espay et al. observed a positive effect of visual cueing on the mean FOGQ score (pre-treatment: 14.2 ± 1.9; post-treatment: 12.4 ± 2.5, *p* = 0.002). In addition, a trend towards an improvement in the frequency of FoG was reported (pre-treatment: 2.92 episodes/day; post-treatment: 2.54 episodes/day, *p* = 0.09). However, Espay et al. reported that two participants did not feel comfortable using the headset (because they were “clunky” or “embarrassing to use in public”) and did not train at home as instructed. Interestingly, Espay et al. did not directly monitor the usage of the system at home. Although, they did request verbally confirmation of compliance, it could be concluded that the independent variable (intervention) was inadequately controlled, meaning the study would fail to meet WWC single-case design standards. Interestingly, participants were instructed to use both visual cueing and auditory feedback. Espay et al. reported that personal preference forced some to rely on only one or none. Four participants favored the combination of visual cueing and auditory feedback; three participants preferred just auditory feedback, three visual cueing alone, and two neither.

In the same year, Bryant and colleagues presented a walking cane device that projected a continuous visual cueing via an attached laser [43]. As the user walked with the walking cane, the laser continuously projected a static horizontal laser line onto the ground at the base of the walking cane. This horizontal laser line was reported to mimic the effect of parallel lines marked on a floor. Although walking canes with attached lasers were available commercially to PwP in 2010, Bryant et al. identified that these devices only projected a red laser line and there was no current evidence on the benefit of different laser line colors on FoG. Therefore, to investigate the effect of laser line colors, Bryant and colleagues presented a walking cane device that projected either a red or a green laser line through an attached commonly available laser pointer.

In a pilot study, Bryant et al. carried out measurements on seven participants (all participants usually used walking aids to ambulate) during two different laboratory-based waking tasks (15.2 m straight walking task with a 180° turn and a 360° turning task) and evaluated the frequency of Off-FoG and On-FoG episodes during each task. For each walking task, participants used the walking cane while it either projected a red laser line, a green laser line or no laser line.

Bryant et al. reported a significant positive immediate effect of a green laser line on both the mean frequency of Off-FoG episodes during the 15.2 m walking task (without laser line: 0.67 ± 0.52; with green laser line: 0.0 ± 0.0, *p* < 0.05) and 360° turning task (without laser line: 2.14 ± 1.35; with green laser line: 0.29 ± 0.49, *p* < 0.05). The effect of a red laser line was negative during the 15.2 m walking task (without laser line: 0.67 ± 0.52; with red laser line: 0.83 ± 0.75, *p* > 0.05) and positive during the 360° turning task (without laser line: 2.14 ± 1.35; with red laser line: 1.43 ± 0.98, *p* > 0.05). When the participants were tested in the On-medicated state, Bryant et al. also reported an immediate positive effect of a green and red laser line on the mean frequency of On-FoG episodes during the 15.2 m walking task (without laser line: 0.29 ± 0.49; with green laser line: 0.0 ± 0.0; with red laser line: 0.14 ± 0.38, *p* > 0.05) and 360° turning task (without laser line: 1.0 ± 1.0; with green laser line: 0.57 ± 0.79; with red laser line: 0.57 ± 0.79, *p* > 0.05). However, due to the small sample size of participants (seven), the generalizability of the results was limited. In addition, it was not reported if analysis of the outcome measures was performed by two assessors, therefore, this study failed to meet WWC single-case design standards.

Bryant et al. suggested that the more positive effect of the green laser line on the frequency of FoG episodes may be due to two factors. Firstly, the general perception that a green light means “go” and a red light mean “stop” as indicated by traffic signals. Secondly, under lighting conditions, the human eye is most sensitive at a wavelength of 555 nm. Therefore, the green color line appeared brighter (approximate wavelength of 532 nm) than the red line (approximate wavelength of 650 nm) during the walking tasks. Using a subjective questionnaire, Bryant et al. also presented data on the participants’ perception of the effectiveness of two laser line colors and observed that most participants believed the green line helped the most during both walking tasks.

**Figure 9:**
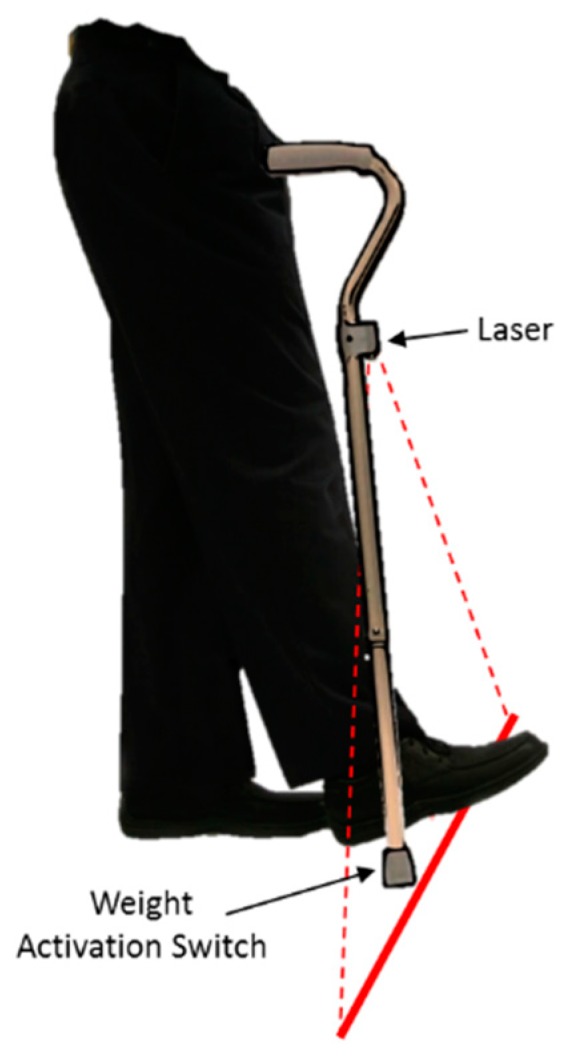
Illustration of the U-Step walking cane. The walking cane device projected a red laser line on the ground only when pressure was applied to the weight activation switch.

In 2011, Donovan et al. described a home-based evaluation of the commercially available U-Step (U-Step Mobility Products, Inc; Skokie, Illinois, USA) walking cane and U-Step walking stabilizer with an attached red laser (Figure 9) [57]. Although these devices were developed outside this review period (2006), Donovan et al. was the first to publish an evaluation of the devices. In contrast to the walking cane presented by Bryant et al., the U-Step walking cane included a weight activated switch that triggered the projection of a static red laser line only when the user placed a downward force on the cane. The U-Step walking stabilizer was a four-wheeled rolling walker with a red laser attached at ankle level. A switch located on the handlebar of the walking stabilizer switched on and off the projection of a static red laser line onto the ground in front of the user.

In an open-label study, Donovan et al. carried out measurements on 26 participants to assess the effect of continuous visual cueing on FoG and the sustainability of the effect over time. During the reported study, participants who normally used a walking cane at home to ambulate received the U-Step walking cane (*n* = 16), participants who normally used a wheeled walker to ambulate received the U-Step walking stabilizer (*n* = 5) and participants who normally used both to ambulate received both the U-Step walking cane and walking stabilizer (*n* = 5). During a baseline assessment period (without the red laser attachment) participants used their walking aids during ambulation for one to two months. Following this period, the U-Step waking cane and the walking stabilizer had the red laser attached. Participants continued to use the devices over a one-month treatment duration. During the treatment period, a FoG questionnaire (FOGQ) was used to evaluate the effect of visual cueing on FoG. Donovan et al. suggested that the FOGQ may be a more relevant outcome measure compared to an assessment of FoG in a doctor’s office. However, it is important to consider that this questionnaire relies on the accuracy of the participant’s ability to recall their FoG severity. Due to an expectation of the treatment benefit the results may have been biased (recall bias).

Over the four weeks treatment period, Donovan et al. observed a significant effect of visual cueing on the mean FOGQ score. The mean reduction in the FOGQ score per-week was: week one: 1.51 ± 0.68 (*p* = 0.036); week two: 1.15 ± 0.67 (*p* = 0.099); week three: 1.73 ± 0.65 (*p* = 0.013); and week four: 0.53 ± 0.43 (*p* = 0.263). Although Donovan et al. identified a trend towards improvement at the end of each of the four weeks, they acknowledged the short duration participants had to use the devices, and therefore their effectiveness during longer periods of use is unknown.

Following Donovan et al. subjective evaluation of the U-Step walking cane and stabilizer, the systems would be objectively evaluated by Bunting-Perry et al. in 2013 [49] and McCandless et al. in 2016 [45]. In a pilot study, Bunting-Perry et al. carried out measurements on 17 participants as they performed laboratory-based walking tasks with and without the projected laser line. In contrast to the previous visual cueing system modalities the walking stabilizer offered On-demand cueing. In this mode of cueing the participant pressed the on/off button during FoG episodes to initiate the laser line and then re-pressed the on/off switch to turn laser off after the FoG episode was aborted.

Bunting-Perry et al. reported an immediate negative effect of visual cueing on the mean frequency of On-FoG episodes during walking task with gait imitation, straight walking, approaching destination and turning FoG triggers (without cueing: 0.94 ± 1.9; with cueing: 1.2 ± 2.3, *p* = 0.84) and walking task with gait imitation, straight walking, approaching destination, turning and passing through doorway FoG triggers (without cueing: 6.2 ± 4.8; with cueing: 7.8 ± 7.8, *p* = 0.58). However, an immediate positive effect was reported during walking tasks with gait imitation, straight walking and approaching destination FoG triggers (without cueing: 2.1 ± 3.6; with cueing: 1.8 ± 3.3, *p* = 0.58). It was suggested by Bunting-Perry et al. that the concurrent use of the walking stabilizer and the self-activation of the laser represents a dual-task that may have reduced the effect of visual cueing. Additionally, Bunting-Perry et al. also suggested that the small sample size of participants and the lack of On-FoG in a quarter of the cohort may have contributed to the null results observed. Although, video footage was analyzed for outcome measures by multiple assessors, inter-assessor agreement was not reported, therefore, this study failed to meet WWC single-case design standards.

In conclusion, Bunting-Perry et al. acknowledged the many pitfalls that exist when studying FoG and provided instructions for future researchers. These included; (1) minimizing dual tasking by using continuous visual cueing; (2) ensuring adequate training with visual cueing devices; (3) test various lasers colors and (4) collect data regarding other features of the participants’ disease, with the goal of characterizing any subgroup that may respond.

To investigate the immediate effect of U-Step walking cane on Off-FoG, McCandless et al. carried out measurements on 20 participants as they performed >3 m laboratory-based walking task with the projected laser line, without the projected laser line, and without the walking cane. McCandless et al. reported an immediate positive effect of visual cueing on the mean percentage of Off-FoG episodes during the walking (without cueing: 40.00 ± 7.85%; with cueing: 27.50 ± 7.34% and without walking cane: 81.58 ± 7.53%). Interestingly, the walking cane without cueing activated showed an immediate improvement compared to no walking cane (baseline), suggesting that a walking cane alone may have a beneficial effect on overcoming FoG. However, as walking tasks only involved two FoG provoking elements, the generalizability of the results during daily activities is limited. Inter-assessor agreement was not documented, therefore, this study failed to meet WWC single-case design standards.

**Figure 10:**
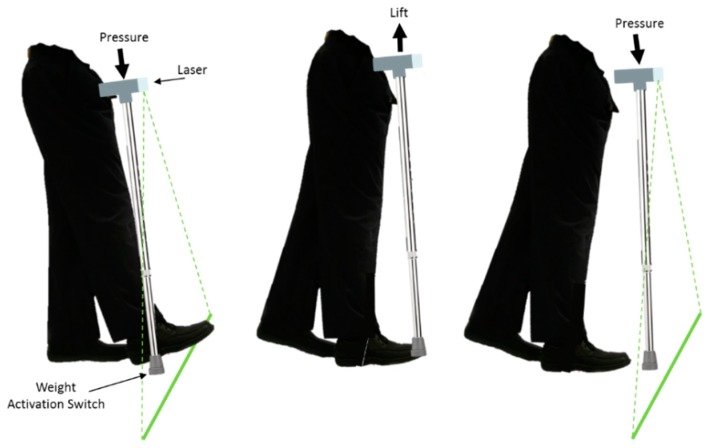
Illustration of the LaserCane, developed at the Chulalongkorn Center of Excellence for Parkinson’s disease and Related Disorders. The LaserCane projected a green laser line on the ground only when pressure was applied to the weight activation switch.

In 2012, Buated et al. presented the LaserCane device that was developed at the Chulalongkorn Center of Excellence for PD and Related Disorders (CCEPDRD) in Thailand (Figure 10) [42]. The LaserCane was similar to the U-Step walking cane and projected static green laser line when the walking cane was pressed down on the ground. With full battery power the cane provided a laser beam of 3 mm in width and 750 mm in length. To investigate the immediate effect of continuous visual cueing on Off-FoG and On-FoG, Buated et al. carried out measurements on 30 participants as they performed a laboratory-based 5 m walking task. During the walking task, participants used the LaserCane while it either projected or did not project a green laser line.

Buated et al. reported an immediate effect of visual cueing on the mean frequency of On-FoG episodes (without cueing: 0.33 ± 0.84; with cueing: 0, *p* < 0.05) and Off-FoG (without cueing: 4.20 ± 6.38; with cueing: 0.76 ± 1.85, *p* < 0.05). Buated et al. also reported that visual cueing was able to reduce FoG in PwP, with greater impact observed among PwP in the moderate Hoehn and Yahr stage (Hoehn and Yahr stage is a commonly used system for describing the progress of PD symptoms) compared to the mild Hoehn and Yahr stage. However, it was not reported if analysis of outcome measures was performed by two assessors, therefore, this study failed to meet WWC single-case design standards.

In 2016, research attention shifted from the integration of visual cueing devices and walking aid devices (walking canes and wheeled walkers), to the development of visual cueing systems that were more wearable and accessible to PwP who do not require the use of walking aids. In this year Zhao et al. proposed to use Google Glass as a platform to evaluate the effect of visual cueing on FoG [48].

**Figure 11:**
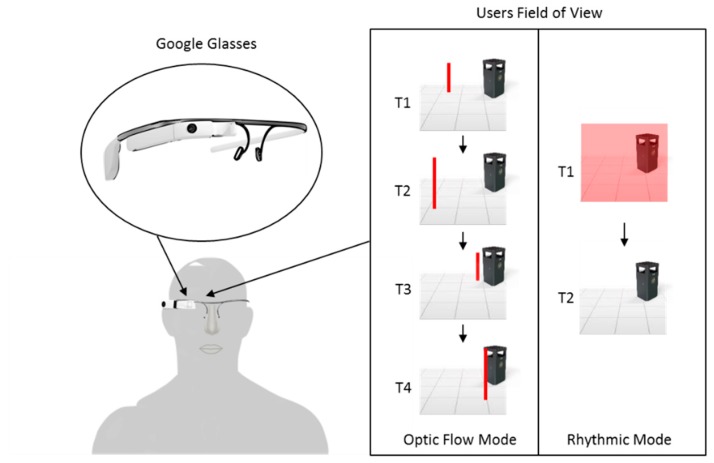
Illustration of user wearing google glass and their field of vision during continuous visual cueing. The Google ‘Glass’ system provides two modes of continuous visual cueing. During optical flow mode, a virtual vertically oriented line continually moves forward on the left and then the right side of the screen at a steady rate (T1–T4). During rhythmic mode, the display flashes on and off a transparent red screen at a set rate (T1–T2).

Zhao and colleagues developed an android application for Google Glass that provided either optic flow or rhythmic visual cueing. Vertically oriented lines on both sides of the Google Glass screen that continually moved forward at a steady rate (ranging from 50 to 150 lines/minute) provided optic flow visual cueing. This type of visual cueing simulated optic flow (pattern of apparent motion in a visual scene) (Figure 11). In contrast, rhythmic visual cueing was provided by rhythmically flashing on and off the Google Glass screen at a preferred steady rate (ranging from 50 to 150 flashes/minute), as illustrated in Figure 11. Although both types of visual cueing have previously been implemented in prototype devices to improve PD gait [29,89], Zhao et al. suggested that implementing them within the Google Glass platform may make visual cueing more accessible for a wider audience. The system allowed the user to interface with the app through voice/gesture control, enabling the adjustment of the optic flow and rhythmic rate by the user.

To investigate the immediate effect of visual cueing on EoD-FoG, Zhao et al. carried out measurements on 12 participants in a laboratory setting. During three 20 m waking tasks and a single ~8 m walking, the frequency and duration of EoD-FoG episodes were evaluated with optic flow visual cueing, with rhythmic visual cueing, and without visual cueing. With all walking tasks considered together, Zhao et al. reported no significant changes in the frequency and duration of EoD-FoG episodes. Although, this study meets WWC single-case design standards, due to the small sample size of FoG episodes (six participants experienced EoD-FoG more than once, four exhibited no EoD-FoG, and two had a single EoD-FoG episode), the effect of visual cueing on EoD-FoG was reported to be inconclusive.

Additionally, Zhao et al. suggest that concentrating on the display while walking potentially created a visual dual-task, which may have reduced the effectiveness of visual cueing. In addition, optic flow visual cueing produced by the system was in constant motion at a fixed rate, independent of the user’s motion. As previously reported, this type of system may have adverse effects on PwP such as dizziness, loss of balance, and even FoG [87].

Using a subjective questionnaire, Zhao and colleagues presented data on the experience of participants who were tested with the device. Four participants found it difficult to synchronize to the continuous visual cueing. They described the continuous visual cueing as annoying, distracting, demanding too much concentration, and hard to see. Conversely, some participants disliked Google Glass’ placement of the visual display in the upper right corner and suggested that images be projected binocularly or more focally in the visual field. Before Google Glass could be adopted for daily use, Zhao et al. acknowledged that there were further possibilities for technical improvements. These included the placement of the display binocularly or towards the center of the visual field to optimize the effects of visual cueing.

In 2017, Tang et al. presented a custom-built wearable device that provided visual cueing via a chest-worn laser that continuously projected either a static or rhythmic horizontal laser line onto the ground one step length in front of the user’s feet [55]. The device was attached over the user’s chest correspond to the sternum with an elastic bandage. In this paper, Tang et al. hypothesized that continuous visual cueing delivered rhythmically compensated for the deficiency of previous reported static visual cueing devices. Flashing on and off the projected laser line at a preferred tempo provided rhythmic visual cueing.

In an experimental study, Tang et al. carried out measurements on 23 participants. During an “8-shaped” laboratory-based waking task, the frequency of On-FoG was evaluated with static visual cueing, with rhythmic visual cueing, and without visual cueing. Tang et al. reported a significant immediate positive effect of rhythmic visual cueing on the frequency of On-FoG episodes (without cueing: 66; with cueing: 23, *p* = 0.001) and immediate positive effect of static visual cueing on the frequency of On-FoG episodes (without cueing: 66; with cueing: 44, *p* = 0.742). Although positive results were shown, Tang et al. acknowledged the limited number of participants tested limited the generalizability of the results. In addition, analysis of outcome measures was reported to be performed by multiple assessors, however, inter-assessor agreement was not reported. Therefore, this study failed to meet WWC single-case design standards.

**Figure 12:**
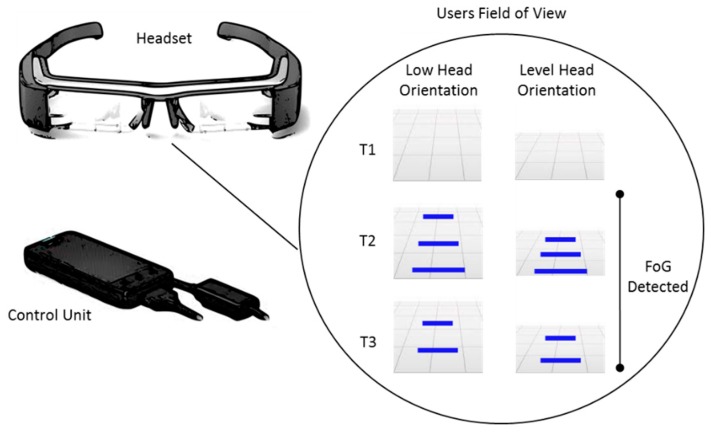
Visual stimuli system presented by Ahn et al. The system consisted of the Moverio BT-200 smart glasses (headset, a control unit and cables) and an android application that displayed parallel blue lines when FoG was detected (T2–T3). As illustrated, these lines move forward in sync to the user’s gait speed and head orientation. For example, as the user lowers their head (to look down) the distance between the lines increases.

Shortly after the Tang et al. publication, Ahn et al. presented the Smart Gait-Aid system (Figure 12) [56]. In contrast to the Zhao et al. Google Glass system, Ahn et al. used the Epson’s (Seiko Epson Corporation. Suwa, Nagano, Japan) Moverio BT-200 smart glasses as a platform to evaluate the effect of On-demand visual cueing on FoG. The BT-200 shared similar features with Google Glass (e.g., accelerometer and gyroscope). However, the BT-200 featured a binocular display that projected images towards the center of the visual field. This feature was important, as Zhao et al. had previously identified limitations of Google Glass’s monocular display [48]. In an innovative step, Ahn and colleagues developed an Android app for the BT-200 that implemented real-time FoG detection and produced visual cueing that adapted to the user’s gait speed and orientation of their head. The adaption of the visual cue to how fast the user walks and their current head direction implied a dynamic system. This was in contrast to the Zhao et al. proposed Google glass system, which provided visual cueing at a rate equal to the user’s average cadence and was not affected by the participant’s current gait speed.

Based on work carried out by Coste et al. [90], who developed a FoG detected algorithm using stride length and cadence, Ahn et al. presented a new a real-time FoG detection algorithm tailored for smart glasses, called ‘FoG Detection On Glasses’ (FOGDOG). By implementing FOGDOG in the Android app, the BT-200 displayed blue lines onto the smart glasses binocular display whenever a FoG episode was identified. The displayed blue lines remained on screen until the user turned them off by touching the BT-200 controller pad. The projected blue lines (0.5 × 0.1 m^2^) give an effect of parallel lines regularly placed on the floor and moved in position based on the downward gaze angle and gait speed of the user. Thus, giving the effect that the lines approached the user while walking. Ahn et al. suggested that such adaptive projection of the projected blue lines on the glasses was critical to maximizing the effectiveness of the system. In a proof of concept study, Ahn et al. carried out measurements on 10 participants to understand the technical effectiveness of their system. While wearing the Smart Gait-Aid system, the duration of FoG episodes was evaluated during five Timed-Up and Go (TUG) tests [91].

By comparing the performance of the FOGDOG to video recordings, Ahn et al. report that their FoG detection algorithm could be employed with a lag time of roughly 1.1 s (time between the appearance of a FoG episode and its detection) at a sensitivity of 97% and specificity of 88%. Ahn et al. reported some limitations of the system, which included the limited computing capability of the smart glasses and battery capacity. To minimize power consumption, Ahn et al. designed the system such that it was automatically activated upon detection of FoG episodes or manually activated by the user depending on the need. However, in spite of such an effort, Ahn et al. observed that the battery capacity was not enough to use the system all day long without recharging the battery. Although Ahn et al. did not report user experience of the system, several participants refused to take part in their experiments due in part to technophobia and that they were doubtful about the effectiveness of visual cueing. In conclusion, Ahn et al. acknowledged there were further possibilities for technical improvements, such as developing a cloud-based solution to offload the image processing task of smart glasses.

**Figure 13:**
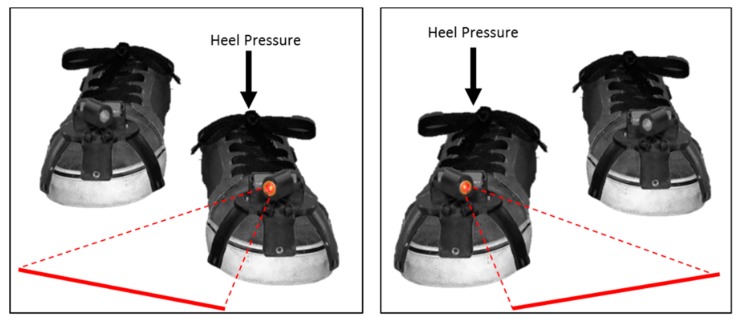
Illustration of the Laser Shoes, developed by Barthel et al. Each laser attachment projected a red laser line in front of the opposite foot when pressure was applied to the heel switch within the shoe.

Furthermore, in 2017, Barthel et al. identified limitations with previous wearable visual cueing devices, which included a lack of ease of application during daily life [51]. Barthel and colleagues reported that the use of smart glasses in daily life poses great challenges on the users’ compliance in wearing such “cumbersome” devices. In an aim to provide a practically acceptable visual cueing device for domestic use, Barthel et al. presented the Laser shoes. The Laser shoes incorporated two laser attachments that were attached to a pair of normal shoes and two heel switches that were placed under each sole of the user’s foot (Figure 13). During gait, each Laser shoe attachment projected a static horizontal red laser line orthogonally in front of the opposite foot during heel strike of said foot. The laser line would remain until the user raises their heel. This cycle repeats itself step after step, delivering visual cueing alternately to each foot.

To investigate the immediate effect of continuous visual cueing on On-FoG and Off-FoG episodes, Barthel et al. carried out measurements on 19 participants as they performed laboratory-based walking tasks with and without visual cueing. Barthel et al. reported a significant immediate positive effect of visual cueing on the percentage of time in Off-FoG (without cueing: 19.6 ± 5.2%; with cueing: 12.9 ± 5.0%, *p* = 0.004) and in On-FoG (without cueing: 8.8 ± 4.1%; with cueing: 6.0 ± 3.1, *p* = 0.004). In addition, Barthel al. also reported a significant immediate positive effect of visual cueing on the frequency of Off-FoG episodes (without cueing: 6.0 ± 1.3; with cueing: 3.3 ± 1.0, *p* = 0.007) and On-FoG episodes (without cueing: 3.3 ± 1.0; with cueing: 2.0 ± 0.9, *p* = 0.028). However, it was reported that video analysis of outcome measures was performed by only one assessor, therefore, this study failed to meet WWC single-case design standards.

Using a subjective questionnaire, Barthel et al. presented data on the experience of the participants who were tested with and without the system. When asked about the efficacy of laser shoes, 12 out of 19 participants reported a moderate to large improvement, four a small improvement, and three no effect. No participants reported a negative impression. Furthermore, 12 out of 19 participants expressed interest in acquiring laser shoes, six were unsure, and one not interested.

### 3.3. Somatosensory Stimuli Devices

**Figure 14:**
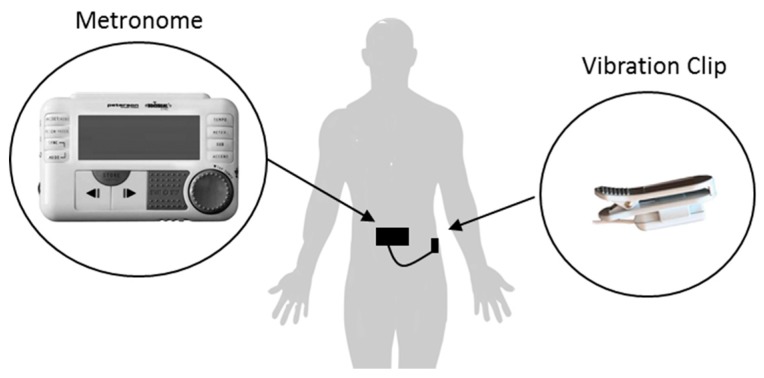
Illustration of a participant wearing the BodyBeat Pulsing Metronome. The metronome was clipped to a belt at the back of the participant and the vibration clip was placed anteriorly over the right side of the pelvis.

In 2016, McCandless et al. purposed the use of the commercially available BodyBeat Pulsing device to investigate the immediate effect of somatosensory cueing on FoG (Figure 14) [45]. The device provided rhythmic tactile stimulation (pulsed vibration) at an adjustable tempo of 10 to 280 beats/minute via an attachable vibration clip. McCandless et al. purposed to attach the vibration clip anteriorly over the right side of the user’s pelvis, so it could be felt easily by the user.

To investigate the immediate effect of somatosensory cueing on Off-FoG, McCandless et al. carried out measurements on 20 participants as they performed >3 m walking task with and without somatosensory cueing. McCandless et al. set the tempo of their device to either 70, 60 or 50 beats/minute, based on participant’s preferences. McCandless et al. reported an immediate positive effect of somatosensory cueing on the mean percentage of Off-FoG episodes during the walking task (without cueing: 81.58 ± 7.53%; with cueing: 68.29 ± 7.25%). However, as walking tasks only featured two FoG provoking elements, the generalizability of the results to daily activities is limited. Although, two experienced neuro-physiotherapists analyzed measurement outcomes, inter-assessor agreement was not documented, therefore, this study failed to meet WWC single-case design standards.

**Figure 15:**
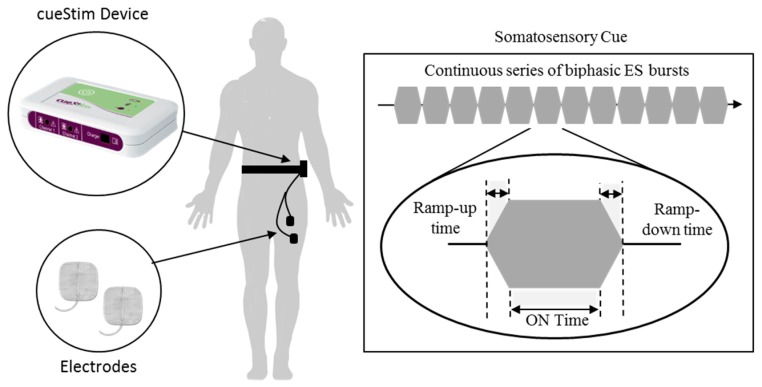
Illustration of a user wearing the cueStim continuous somatosensory cueing device. The device was worn on the waist and delivered a continuous series of biphasic electrical stimulation (ES) bursts through the use of 50 × 50 mm^2^ skin surface electrodes placed over the skin surface of the hamstring or quadriceps muscle of the body side most affected by the user.

In 2018, Rosenthal et al. proposed the use of sensory electrical stimulation to provide somatosensory cueing [54]. In this paper, Rosenthal et al. hypothesized that somatosensory cueing delivered at a fixed tempo would modify the users walking patterns and help to alleviate On-FoG. This hypothesis was based on previously reported mechanisms responsible for the positive effects of cueing, such as step synchronization and enhanced proprioceptive information processing [33,36]. To investigate the hypothesis Rosenthal et al. used a custom-built electrical stimulator, cueStim. The cueStim device (105 × 65 × 19 mm^3^, 100 g) was a Bluetooth enabled voltage-controlled two-channel electrical stimulator developed within the REMPARK (FP7 project REMPARK ICT-287677) project. The device was worn on the waist and delivered a continuous series of electrical stimulation (ES) bursts through the use of PALs 50 × 50 mm^2^ skin surface electrodes placed on the skin surface of the hamstring or quadriceps muscle of the body side most affected by PD (Figure 15). In its then configuration, each ES burst was delivered at a rate of 86 burst/minute and consisted of 100 ms ramp-up time, 500 ms On time, 100 ms ramp-down time and 0 ms Off time. The amplitude of the stimulation burst was adjusted for each participant (using a smartphone application design to communicate with the cueStim device) such that a sensory response was elicited but that the amplitude was not of sufficient intensity to produce a motor response.

To investigate the effect of continuous somatosensory cueing, Rosenthal et al. carried out home-based measurements on nine participants as they performed a self-identified walking task with and without somatosensory cueing. The walking task aimed to reproduce daily activities which usually elicited FoG episodes. Rosenthal et al. reported an immediate statistically significant positive effect of somatosensory cueing on the reduction on On-FoG (58.28 ± 33.89%). However, it was reported that analysis of outcome measures was only performed by one assessor, meaning the study failed to meet WWC single-case design standards.

**Figure 16:**
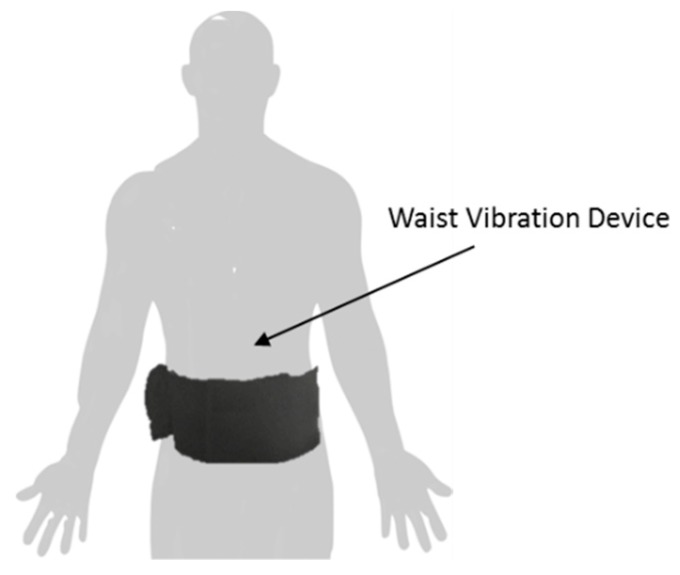
Illustration of a user wearing the continuous somatosensory cueing device developed by Gonçalves et al. Four haptic drivers/vibration units were placed inside the waistband and arranged with an intermediate spacing (15 cm) that facilitated a 76 to 110 cm waist size, while providing the stimulation to the navel, spine, right side and left side of the waist. The waistband was designed to be robust enough to support the electronics (overall weight of 458 g).

In the same year, Gonçalves et al. focused on investigating the optimal vibration frequency and minimum duration of vibration to be used in a somatosensory system for PwP, to help them to overcome FoG [92]. To investigate the hypothesis, Gonçalves et al. developed a custom-built somatosensory cueing system (Figure 16). The system was encapsulated in a waistband and provided tactile stimulation (pulsed vibration) at the navel, spine, right side and the left side, allowing the system to meet the previously reported requirements for developing a robust, functional, ergonomic and wearable system to provide vibration cueing for addressing FoG [93]. The processing unit was developed on the Arduino Mega 2560 platform and controlled four vibration units through the Adafruit Industries’ DRV2605 haptic drivers. The vibration units were mini vibration motors 2.0 mm (Seed Studio Electronic), a special type of coin vibration motor, also known as “pancake” vibrator motors and provided vibration at a frequency range of 60–300 Hz, with an amplitude of 0.2–2.8 G. The system was Bluetooth enabled, and both smartphone and desktop graphical interfaces were developed to allow the wireless configuration of cueing parameters (frequency and duration of vibration). A single 12 V Lithium-Ion battery powered the system.

Although Gonçalves et al. did not evaluate the performance of their system on ameliorating FoG, they did investigate the optimal vibration frequency and minimum duration of vibration required to perceive best cueing. Based on previous works, Gonçalves et al. concluded that a vibration frequency range between 80 and 250 Hz must be considered and carried out measurements on 15 healthy and 15 PwP participants. Although, it has been observed that PwP present a lower perception than healthy subjects, Gonçalves et al. reported that it was possible to conclude that on average the frequency by which all subjects present a high vibration sensitivity at waist body level, was 180 Hz. It was also observed that greater than 250 ms of vibration duration was detectable by almost all PwP and the healthy subjects. This was reported to be the first step of implementing their system to help PwP ameliorating FoG. Gonçalves et al. also reported that participants did not consider the use of the waistband uncomfortable and considered it possible to perform their daily tasks while receiving somatosensory cueing.

**Figure 17:**
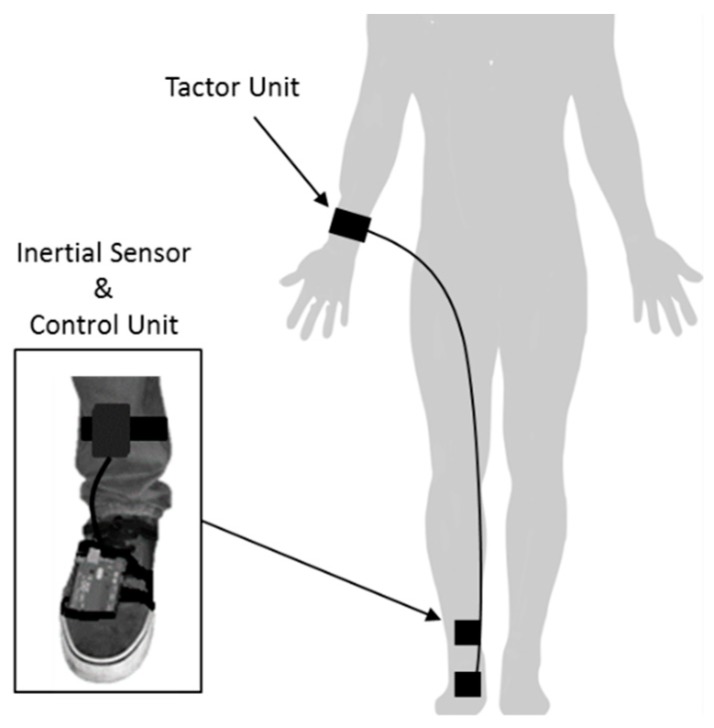
Illustration of a user wearing the continuous somatosensory cueing device VibroGait, developed by Mancini et al. A haptic drivers/vibration unit was placed on wrist and connected to a control unit and inertial sensor.

In 2018, Mancini et al. further proposed the use of vibration to provide somatosensory cueing [94]. In this paper, Mancini et al. hypothesized that enhancing proprioceptive stimuli, in the form of tactile stimulation, may be useful in improving sensory integration and therefore alleviating FoG in PwP. To test the hypothesize Mancini et al. used the VibroGait, a wearable somatosensory cueing system, previously described in [95] (Figure 17). The system plugged into inertial sensors placed on the shins of the user and consists of a novel controller unit (Arduino microcontroller) that senses through a gyroscope when the foot is on the ground and activates the tactor unit to generate a vibration. The tactor was a C-2 tactor (Engineering Acustic, Inc.) with a primary resonance in the 200–300 Hz range. When a pulse width modulation sine-wave was sent from the control unit into the tactor, the “contactor” oscillated perpendicular to the user’s skin. As explained by Mancini et al., this created a “point-like sensation that is easily felt and localized”. The vibration intensity was reported to be similar to that of a cell phone operating in vibration mode.

To investigate the immediate effect of continuous somatosensory cueing on Off-FoG episodes, Mancini et al. carried out measurements on 25 participants as they performed laboratory-based walking tasks with and without somatosensory cueing. The following measures were extracted to objectively characterize FoG using inertial sensors and were previously described and validated in Mancini et al. [96]; (1) FoG ratio as index of freezing severity calculated as the power spectral density ratio between high (3–8 Hz) and low (0–3 Hz) frequencies of antero-posterior shin accelerations; and (2) the percentage of time spent in FoG during the task, calculated as the time in which the FoG ratio was higher than 1 (for either right or left foot). Mancini et al. reported an immediate positive effect of somatosensory cueing on the percentage of time in Off-FoG during cueing (single task: 19 ± 18%, *p* = 0.5; dual task: 18 ± 15%, *p* = 0.2). The majority of cueing studies to date validate FoG measurement outcomes, such as the number and duration of episodes subjectively, using experienced clinical judgment. Interestingly, this study analyzed outcome measures through a sensor-based FoG detection algorithm. Although this algorithm’s calculated FoG ratio has been previously shown to correlate with a subjective clinical assessment of FoG severity [96], in the current study by Mancini et al. [94], the results presented only reflected the percentage of time in FoG. To the authors’ knowledge the analyses of a correlation between the algorithm’s objective measure of the percentage of time in FoG and a subjective measure of the percentage of time in FoG by two assessors (with an inter-assessor agreement) was not performed. Therefore, we would conclude that this study failed to meet WWC single-case design standards.

Results from subject impressions on the efficacy of somatosensory cueing on FoG while turning based on a Likert scale showed that ~50% of the participants reported an improvement in FoG using closed-loop cues

## 4. Discussion

This paper aimed to provide a systematic review of current cueing systems focusing on systems with the potential to be used at home as a self-administer intervention. Both system technical characteristics and the system efficacy are described. Of the 18 papers included in this review, 18 different cueing systems have been identified, of which eight of them (44%) have been published since 2015, indicating that this is an expanding field of study. The general overview of the reviewed cueing systems is presented in Table 2, Table 3 and Table 4. These tables highlight the key technical characteristics of all the identified cueing systems.

Since 2010, auditory cueing systems have evolved from the development of custom-built technologies (as presented by Bachlin and Arias) to the use of stand-alone commercial devices (i.e., digital metronomes), to the integration of custom-built devices (i.e., movement sensors) with commercially available units such as smartphones and smartglasses. Similarly, visual cueing systems have evolved from custom-built technologies to the use of stand-alone commercial devices (i.e., the U-Step Lasercane and the U-Step walking stabilizer), and the integration of custom-built Android applications with commercially available smartglasses (i.e., Google Glass and Epson BT200). Somatosensory cueing systems have not had the time to evolve in parallel with auditory or visual cueing systems as a method to ameliorate FoG. However, both novel custom-built technologies and stand-alone commercial devices (i.e., digital vibrating metronome) have been utilized to investigate somatosensory cueing.

From the 18 identified cueing systems, five of them provided auditory cueing, seven of them provided visual cueing, three of them provided somatosensory cueing and a further three provided dual cueing modalities (two auditory and visual cueing systems and one auditory and somatosensory cueing system). Auditory cueing has been shown to ameliorate EoD-FoG episodes [44,48], Off-FoG episodes [45] and On-FoG episodes [75]. Visual cueing has been shown to ameliorate Off-FoG episodes [42,43,45,51] and On-FoG episodes [42,43,49,51,55]. The effect of somatosensory cueing has also been shown to ameliorate Off-FoG episodes [45,94] and On-FoG episodes [54]. However, due to the limited number of studies [45,48] that directly compared different cueing modalities, further investigation will be required to establish which modality is the most effective in different circumstances. 

Cueing systems have been described that are capable of providing Continuous or On-demand cueing. Of the 18 identified cueing systems, 13 of them (72%) have been characterized as Continuous cueing systems, five of them (28%) as On-demand cueing systems. Continuous cueing systems provide cueing continuously even if not required and predominantly aim to prevent FoG from occurring. An advantage of Continuous cueing systems is the simple technological requirements of the system and the ease of application, which typically consists of a single device (i.e., digital metronome or lasercane). However, as a result of the continuous delivery of the stimulus, these systems may prove to be less effective during extended periods of usage due to possible habituation and compliance issues.

Due to the identified weaknesses of Continuous cueing, On-demand cueing was proposed and provides cueing only when required (during a FoG episode) and predominantly aims to relieve FoG episodes by reducing their duration. In comparison to Continuous cueing systems, On-demand systems are more sophisticated and predominantly incorporate a FoG detection algorithm [56,71,82,85]. A key characteristic of On-demand cueing systems relates to the sensitivity and specificity and lag time (time between the appearance of a FoG episode and its detection) parameters of its FoG detection algorithm. These parameters define the performance of the algorithm. However, it is unclear which parameters are most important to the overall efficacy of the systems. In 2010, Bachlin and colleagues implemented a real-time FoG detection algorithm with an average sensitivity of 73.1% and specificity of 81.6% at a lag time >2 s [75]. For some patients, this low specificity value resulted in the patients requesting to hear the auditory cueing less often, and for some patients, the low sensitivity value resulted in the patients requesting to hear the auditory cueing more often and was reported to affect the overall acceptance of the system. Interestingly, Bachlin et al. suggested that sensitivity and specificity are equally important [72], while more recently, Kwon et al. reported that sensitivity is more important than specificity, suggesting that low false negatives matter more than low false positives [97]. However, due to refinements in FoG detection algorithm designs and increased processing power of some cueing systems, recent On-demand cueing systems have reported sensitivity values of 82.2% [82], 97% [56] and 97.1% [85]. In addition, specificity values of 92.8% [82] and 88% [56] were achieved. The cueing system presented by Mazilu et al. operated at a lag time of 0.5 s [85], this was a significantly lower lag time than previous systems 1.1 s [56] <2 s [75] and 3.2 s [82]. However, a short lag time typically results in reduced specificity. The need for such a short lag time is up for discussion, and it may be suggested that providing cueing for FoG episodes that last only a short time may not be required, as these FoG episodes may not be troublesome for PwP.

Interestingly, Bunting-Perry et al. evaluated an On-demand cueing system that was self-activated. Unlike other On-demand cueing systems the user activated/deactivated cueing by pressing a button, therefore the sensitivity and specificity and lag time of the system was dependent only on the user’s ability to detect FoG and press a button. However, the concurrent use of the walking stabilizer and the self-activation of cueing was suggested to represent a dual-task that may reduce the effect of self-activated cueing.

Whether Continuous cueing is more effective than On-demand at ameliorating FoG, or vice versa, remains to be established. Continuous cueing systems have been shown to reduce the frequency of EoD-FoG episodes [44,48], Off-FoG episodes [42,43,45,51], and On-FoG episodes [42,45,51,54,55]. Interestingly, Zhao et al. demonstrated that their Continuous cueing system was only effective at reducing the frequency of EoD-FoG episodes while the PwP were performing turning maneuvers with auditory cueing and had no effect during other walking scenarios [49]. In the same study, it was also shown that the Continuous cueing system did not affect the duration of EoD-FoG episodes. However, Arias and Cudeiro demonstrated that their Continuous auditory cueing system might have a positive effect on the duration of EoD-FoG episodes [44]. In comparison, On-demand cueing systems have been shown, in some cases, to reduce the duration and frequency of FoG episodes for some PwP. However, results are limited and based on small participant cohorts [49,75,85].

Another key characteristic of cueing systems is the cueing stimulus and the information that it presents. In the last decade, cueing systems have used three different types of cueing stimuli; rhythmic, static and optic flow. These types of stimuli may provide temporal, spatial, and/or proprioceptive information and aim to ameliorate FoG through different mechanisms, which remains to be established. Of the 18 identified cueing systems, nine of them (50%) have been characterized as rhythmic cueing systems, five of them (28%) as static systems, two of them (11%) as optic flow systems, and two of them (11%) as dual systems (one rhythmic/static and one rhythmic/optic flow).

Auditory and somatosensory cueing systems have predominantly provided rhythmic cueing stimuli. These systems deliver cueing rhythmically at a set tempo that is either fixed [54,75], adjustable [44,45,49,82,85,92] or dynamic [94]. Auditory stimuli have been provided in the form of sounds at a given frequency and duration. Similarly, somatosensory stimuli have been provided in the form of vibrations [45,92,94] or electrical stimulation [54] at a given frequency and duration. However, detailed information on the stimulus produced was not presented for all systems. The importance of presenting details of the stimuli used should not be overlooked as recent evidence suggests that improvements to gait in PwP are directly influenced by the specific nature of cueing stimuli [59,98].

The majority of visual cueing systems have used static cueing stimuli [42,43,45,49,51,55]. However, a limited number of systems have provided optic flow [48,56,61] or rhythmic [48,55] cueing stimuli. A static visual stimulus has been provided in the form of a horizontal laser line projected on the ground. Interestingly, Bryant et al. demonstrated that the color of the laser line may affect the efficacy of the cueing systems [43]. It was suggested that a green laser line was more effective than a red laser line at ameliorating FoG. In an alternative method of visual cueing, an optical flow visual stimulus has been provided through smartglasses in the form of moving virtual checkboards [61], moving vertical red lines [48] or moving horizontal blue lines [56] that may enhance the perception of movement. In a similar fashion to rhythmic cueing stimuli, these systems deliver cueing at a set tempo that was either adjustable [48] or dynamic [55,61]. A rhythmic visual stimulus has been provided through a laser cane in the form of a flashing horizontal laser line projected on the ground [55] or through smartglasses in the form of a virtual flashing screen [48]. The most effective type of visual cueing stimuli remains to be established. In fact, considering that the majority of the cueing systems identified in this review were evaluated in a laboratory setting, with limited results often reported, many questions remain on the effectiveness of such systems to ameliorate FoG in a home setting and subsequently improve QoL for PwP.

It remains to be seen which characteristic of cueing systems will prove to be most appropriate in the long-run. This is perhaps expected for a growing field of research and may be due to experimental nature of the cueing studies and the lack of a gold standard for evaluating the effect of cueing systems on FoG, which resulted in the adoption of various cueing technologies and evaluation methodologies making comparisons between systems difficult.

The limited number of statistically significant findings is disappointing. For example, only Arias et al., Barthel et al., Buated et al., Bryant et al., McCandless et al., Rosenthal et al. and Tang et al. reported statically significant improvements in outcome measures. Additionally, only of one the studies (Zhao et al.) passed the WWC quality test for single case study design, indicating that more attention and rigor needs to be applied to the design of studies used to evaluate these technologies.

Another consideration is the cost and usability of these cueing devices. The cost of electronics hardware continues to come down and when compared to possible surgical interventions, the cost of these systems should not be a significant barrier. The usability of these systems is however a very important consideration and can be best achieved with user-centered design approaches, where the user community is a key stakeholder in the design process. Previous work by our group has shown that good usability can be achieved with older adults using this approach [99,100,101,102].

There is potential for future work to exploit to full benefit of cueing for FoG in PD, through the use of new technologies as they emerge. While it is challenging to perform the kind of clinical evaluations described in this review, there is a particular requirement for more thorough validation of these systems as much work to date has involved low participant numbers, with studies carried out in laboratory environments rather than home environments. There is also scope in future studies for enhanced experimental design so that the robustness of the evidence being produced is enhanced.

## Figures and Tables

**Table 1:** Freezing of Gait (FoG) subtypes and response to dopaminergic medication.

FoG Subtype	Off-Medication State	On-Medication State
Dopaminergic-Responsive FoG	FoG Occurs	No FoG
Dopaminergic-Induced FoG	No FoG	FoG Occurs
Dopaminergic-Resistant FoG	FoG Occurs	FoG Occurs

**Table 2:** Overview of auditory cueing devices.

Ref.	Cueing Technology	Cueing Mode	Cueing Trigger	Cueing Stimuli Type, Sound (Frequency, Duration)	Cueing Tempo Configuration	Effectiveness
[75]	Custom-built: Small Bluetooth enabled Linux based wearable computer and Bluetooth enabled movement sensor. Commercial: Wired headphones.	O.	FoG detection algorithm (73.1%, 81.6%, <2 s) ^1^.	Rhythmic, ticking (NR, NR).	Fixed (60 bpm).	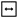 Frequency and duration of Off-FoG ^4^. ↓ Frequency and duration of Off-FoG ^5^. ↓ Frequency of On-FoG ^4^. ↓ Duration of On-FoG ^3^. ↑ Duration of On-FoG ^3^.
[44]	Custom-built: Metronome. Commercial: Headphones.	C.	NA.	Rhythmic, tone (4625 Hz, 50 ms).	Adjustable (NR).	↓ Frequency of EoD-FoG. ↓ Duration of EoD-FoG.
[50]	Commercial: Metronome (SDM300 SAMICK).	C.	NA.	Rhythmic, tone (45–440 Hz, NR).	Adjustable (40–216 bpm).	↑ Frequency of Off-FoG.
[45]	Commercial: Metronome (Peterson BodyBeat).	C.	NA.	Rhythmic, rimshot, clave, wood Block or beep (NR, NR).	Adjustable (10–280 bpm).	↓ Percentage of Off-FoG per walking task.
[82]	Custom-built: FoG detection device and Android application. Commercial: Smartphone and wireless ear-set.	O.	FoG detection algorithm (82.2%, 92.8%, 3.2 s) ^1^.	Rhythmic, ticking, musical beats, clapping or verbal (NR, NR).	Adjustable (NR).	NR.
[85]	Custom-built: Two movement sensors devices and Android application. Commercial: Smartphone and wired headphones. (GaitAssist)	O.	FoG detection algorithm (97.1%, 26.5%, 0.5 s) ^2^.	Rhythmic, ticking (NR, NR).	Adjustable (NR).	↓ Frequency of FoG. ↓ Duration of FoG.
[48]	Custom-built: Android application. Commercial: Google Glass.	O.	NA.	Rhythmic, NR (NR, NR).	Adjustable (NR).	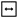 Frequency and duration of EoD-FoG. ↓ Frequency of EoD-FoG ^6^.

NR = not reported; NA = not applicable; C = Continuous; O = On-demand; ↓ = reduced; ↑ = increased; 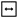 = no change. ^1^ sensitivity, specificity, lag time. ^2^ sensitivity, false negative, lag time. ^3^ For one participant. ^4^ For two participants. ^5^ For three participants. ^6^ Only during complex 360° turns.

**Table 3:** Overview of visual cueing devices.

Ref	Cueing Technology	Cueing Mode	Cueing Trigger	Cueing Stimuli Type, Visual (Color)	Cueing Tempo Configuration	Effectiveness
[61]	Custom-built: Binocular Smartglasses. (Visual-auditory walker)	C.	NA.	Optical Flow, moving virtual checkerboard-tiled (NR).	Dynamic (Automatically adjusts to gait speed).	↓ FOGQ score.
[43]	Custom-built: Walking cane with attached laser.	C.	NA.	Static, horizontal laser line (green or red).	NA.	↓ Frequency of On-FoG and Off-FoG (green laser). ↓ Frequency of On-FoG (red laser). ↑↓ Frequency of Off-FoG (red laser). ^1^
[45,57]	Commercial: U-Step walking cane.	C.	Weight-activated switch.	Static, horizontal laser line (red).	NA.	↓ Percentage of Off-FoG per walking task.
[49,57]	Commercial: U-Step walking stabilizer.	O.	Push button switch.	Static, horizontal laser line (red).	NA.	↑↓ Frequency of On-FoG. ^2^
[42]	Custom-built: Walking cane with attached laser. (LaserCane)	C.	Weight-activated switch.	Static, horizontal laser line (green).	NA.	↓ Frequency of On-FoG and Off-FoG steps
[48]	Custom-built: Android app. Commercial: Google Glass monocular smartglasses.	C.	NA.	Optical Flow or Rhythmic, moving virtual vertically oriented lines or flashing virtual screen (red).	Adjustable.	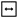 Frequency and duration of EoD-FoG.
[55]	Custom-built: Chest worn laser.	C.	NA.	Static or Rhythmic, horizontal laser line or flashing horizontal laser line (NR).	Adjustable.	↓ Frequency of On-FoG.
[56]	Custom-built: Android app. Commercial: Epson’s Moverio BT-200 binocular smartglasses. (Smart Gait-Aid)	O.	FoG detection algorithm (97%, 88%, 1.1 s).	Optical Flow, moving virtual horizontal lines (blue).	Dynamic (Automatically adjusts to gait speed).	NR.
[51]	Custom-built: Shoe attachment with laser (Laser Shoes).	C.	Heel strike switch.	Static, horizontal laser line (red).	NA.	↓ Frequency of On-FoG and Off-FoG. ↓ Percentage of time in On-FoG and Off-FoG.

NR = not reported; NA = not applicable; C = Continuous; O = On-demand; ↓ = reduced; ↑ = increased; 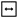 = no change. ^1^ Negative effect during 15.2 m task and positive effect during turning task. ^2^ Negative effect during walking task 1 and 2 and positive effect during walking task 3.

**Table 4:** Overview of somatosensory cueing devices.

Ref.	Cueing Technology	Cueing Mode	Cueing Trigger	Cueing Stimuli Type, Somatosensory (Frequency, Duration)	Cueing Tempo Configuration	Effectiveness
[45]	Commercial: Metronome (Peterson BodyBeat).	C.	NA.	Rhythmic, vibrations (NR, NR).	Adjustable (10–280 bpm).	↓ Percentage of walking task with Off-FoG.
[54]	Custom-built: Two channel electrical stimulator (cueStim).	C.	NA.	Rhythmic, Biphasic electrical pulses (NR, 500 ms).	Fixed (86 bpm)	↓ Frequency of On-FoG.
[92]	Custom-built: Vibrating waistband.	C.	NA.	Rhythmic, vibrations (NR, 100–1000 ms).	Adjustable (80–250 Hz).	NA
[94]	Custom-built: Vibrating system (VibroGait).	C.	NA.	Rhythmic, vibrations (200–300 Hz, NR)	Dynamic (Automatically adjusts to gait speed).	↓ Frequency of and time spent in Off-FoG.

NR = not reported; NA = not applicable; C = Continuous; ↓ = reduced.
